# Emergence of Highly Pathogenic Avian Influenza A Virus (H5N1) of Clade 2.3.4.4b in Egypt, 2021–2022

**DOI:** 10.3390/pathogens12010090

**Published:** 2023-01-05

**Authors:** Zienab Mosaad, Mohamed H. Elhusseiny, Ali Zanaty, Mustafa M. Fathy, Naglaa M. Hagag, Wesam H. Mady, Dalia Said, Moataz M. Elsayed, Ahmed M. Erfan, Neveen Rabie, Abdelhafez Samir, Mohamed Samy, Abdel-Satar Arafa, Abdullah Selim, Ali M. Abdelhakim, Johanna F. Lindahl, Samah Eid, Åke Lundkvist, Momtaz A. Shahein, Mahmoud M. Naguib

**Affiliations:** 1 Reference Laboratory for Veterinary Quality Control on Poultry Production, Animal Health Research Institute, Agriculture Research Center (ARC), Giza 12618, Egypt; 2 Animal Health Research Institute-Mansour Branch, Agriculture Research Center (ARC), Dakahlia 35511, Egypt; 3 General Organization for Veterinary Services, Giza 12618, Egypt; 4 Zoonosis Science Center, Department of Medical Biochemistry and Microbiology, Uppsala University, 75121 Uppsala, Sweden

**Keywords:** avian influenza, HPAI, H5N1, wild migratory birds, clade 2.3.4.4b, Egypt

## Abstract

Wild migratory birds have the capability to spread avian influenza virus (AIV) over long distances as well as transmit the virus to domestic birds. In this study, swab and tissue samples were obtained from 190 migratory birds during close surveillance in Egypt in response to the recent outbreaks of the highly pathogenic avian influenza (HPAI) H5N1 virus. The collected samples were tested for a variety of AIV subtypes (H5N1, H9N2, H5N8, and H6N2) as well as other pathogens such as NDV, IBV, ILT, IBDV, and WNV. Among all of the tested samples, the HPAI H5N1 virus was found in six samples; the other samples were found to be negative for all of the tested pathogens. The Egyptian HPAI H5N1 strains shared genetic traits with the HPAI H5N1 strains that are currently being reported in Europe, North America, Asia, and Africa in 2021–2022. Whole genome sequencing revealed markers associated with mammalian adaption and virulence traits among different gene segments, similar to those found in HPAI H5N1 strains detected in Europe and Africa. The detection of the HPAI H5N1 strain of clade 2.3.4.4b in wild birds in Egypt underlines the risk of the introduction of this strain into the local poultry population. Hence, there is reason to be vigilant and continue epidemiological and molecular monitoring of the AIV in close proximity to the domestic–wild bird interface.

## 1. Introduction

Wild migratory birds serve as reservoirs for several pathogens that can further spread to other domestic birds [1,2]. Infectious bronchitis virus (IBV) [3,4,5], Newcastle disease virus (NDV) [3,6,7], and avian influenza virus (AIV) [6,7] are common avian viruses circulating in migratory birds. In addition, a previous study showed moderate exposure of wild birds to infectious bursal disease virus (IBDV) [8]. The highly pathogenic avian influenza (HPAI) virus of the H5Nx subtype has been circulating in wild migrating birds over the last two decades and has become endemic in domestic poultry in several countries [9,10,11,12]. Since the 1990s, avian influenza H5 strains have shown a distinct evolutionary pattern by accumulating mutations and reassortment with other AIV subtypes [13,14] and evolved into 10 clades (0–9) and several subclades based on the phylogenetic character of its hemagglutinin (HA) gene segment [15].

In 2005, HPAI H5N1 of clade 2.2.1 hit several countries and became endemic in a few [16]. Later in 2014, HPAI H5N8 of clade 2.3.4.4a caused several outbreaks in both wild and domestic birds in Europe [17]. A new wave was observed in 2016–2017 by a novel reassortant of H5N8 and H5N5 of clade 2.3.4.4b and resulted in the death of millions of birds in several countries worldwide [18]. Recently, in late 2020, different genotypes of HPAI H5N1 within clade 2.3.4.4b have emerged in wild birds and were detected in several countries in Africa, Asia, Europe, and North America [19,20,21,22], which led to the death of over 70 million birds [21,23]. During natural circulation, the HA gene of the HPAI H5N8 clade 2.3.4.4b reassorted with four different low pathogenic viruses to produce the recent H5N1 virus, which then reassorted with other distinctive AIV variants and generated at least 15 different H5N1 genotypes [24]. In addition, these H5N8 variants mixed with other avian influenza subtypes to create new H5N2, H5N3, H5N4, and H5N5 variants [14,25]. The HPAI H5N1 clade 2.3.4.4b is now the most common strain responsible for global avian influenza outbreaks [24] and has been reported in a wide range of host species including wild birds, domestic poultry, and wild mammals, indicating an increased risk for virus incursion into new hosts [25]. Most recently in 2022, two human infections were reported in Spain [25] and the United Kingdom [26].

In Egypt, the HPAI H5N1 virus was first reported in 2006 within clade 2.2.1 and became endemic among the poultry population for over a decade [27,28]. In 2016, the HPAI H5N8 virus of clade 2.3.4.4b was detected in Egypt in migratory birds [29,30,31,32,33]. Within a short period of time, several strains of HPAI H5N8 were found in domestic poultry populations in many Egyptian governorates, posing a serious impact on the poultry sector [31,34,35,36]. In 2018/2019, the HPAI H5N8 virus was also engaged in the formation of a novel HPAI H5N2 strain in Egypt via reassortment with the local low pathogenic avian influenza (LPAI) H9N2 virus [37,38]. According to epidemiologic data, the Egyptian H5N1 clade 2.2.1.2 has been displaced by the HPAI H5N8 clade 2.3.4.4b, making it the most frequently reported H5 subtype in the Egyptian poultry sector [39].

The current study aimed to monitor the avian influenza virus in wild migratory birds in Egypt during 2021–2022 in response to the recent HPAI H5 outbreaks in Europe and Asia. The analyses performed on the collected samples included the detection and isolation of six HPAI H5N1 strains in different wild migratory bird species in Egypt, and the genetic and phylogenetic characterizations of these strains are provided.

## 2. Materials and Methods

### 2.1. Sample Collection

Oropharyngeal swabs and tissue samples were collected from 190 wild migratory birds from four governorates in Egypt between October 2021 and March 2022 (Table 1). These birds were initially caught by residents of the same governorate by nest trapping and were placed in cages for trading purposes. Upon examination, the birds were found apparently healthy, sick, or dead, and clinical symptoms were recorded. Oropharyngeal swabs of live birds were performed by authorized veterinarians and available precautions were taken. Birds suffering from severe symptoms including not being able to eat or drink were euthanized by cervical dislocation and transported to the Animal Health Research Institute (AHRI), Giza, Egypt, and postmortem inspection was performed on freshly dead birds (Table 1). Tissues including brain, liver, pancreas, kidney, lung, and trachea were obtained, homogenized, suspended in sterile PBS, and cleared by centrifugation for 15 min at 3000 rpm and 4 °C [40]. Tissues from each bird were pooled together and treated as one sample. Ethical approval for sample collection and postmortem examination was obtained from the Review Board of AHRI-Egypt under number AHRI-202232.

### 2.2. Virus Detection and Isolation

For each collected sample, RNA was extracted from the supernatant liquid using the QIAamp Viral RNA Mini Kit (Qiagen, Hilden, Germany) following the manufacturer’s instructions. Using specific primers and probes listed in Supplementary Table S1, the RNA was examined against the matrix (M) gene of influenza A viruses [41]. Positive samples were further tested for H5, H6, and H9 as well as N1, N2, and N8 by real-time reverse transcriptase polymerase chain reaction (RT-qPCR) (Supplementary Table S1) [42,43,44]. Furthermore, all samples were screened for NDV, IBV, ILT, IBDV, and West Nile virus (WNV) to explore the possibility of infection with other viruses [45,46,47,48]. Reaction mixtures were prepared using the RT-qPCR Verso 1-step™ Real-Time PCR Kit (Thermo Fisher Scientific, Waltham, MA, USA, Catalog no. AB4101A) and performed using a Stratagene MX3005 real-time PCR machine (Agilent Technologies, Santa Clara, CA, USA).

For each positive sample, 0.1 mL of the supernatant fluid was injected into three separate specific pathogen-free embryonated chicken eggs of 9–11 days of age. The inoculated eggs were then incubated at 37 °C and monitored daily for two days, after which allantoic fluid was retrieved from the eggs, and tested for virus hemagglutination activity by the HA assay [49].

### 2.3. Nucleotide Sequencing and Phylogenetic Analyses

The HA and NA genes of all positive samples were amplified. Furthermore, based on the results of the HA gene sequences, whole genome of the A/Ibis/Egypt/RLQP-229S/2022 isolate was performed using specific primers as described in Supplementary Table S2. The PCR was carried out using an Applied Biosystems thermal cycler (ProFlexTM PCR System) using an Easyscript One-Step RT-PCR Kit (TransGen Biotech, Beijing, China). Size-specific PCR products for each gene were separated by gel electrophoresis and purified using the QIAquick Gel Extraction Kit (Qiagen, Hilden, Germany).

Purified products were used for nucleotide sequencing using the BigDye Terminator v3.1 Cycle Sequencing Kit (Applied Biosystems, Waltham, MA, USA) and purified using a Centrisep spin column, (Thermo Fisher, Waltham, MA, USA). Sequencing was performed using an ABI 3500xL Genetic Analyzer (Applied Biosystems, Waltham, MA, USA). Generated sequences from this study were deposited in the GenBank database under accession numbers provided in Supplementary Table S3. The N-linked glycosylation pattern on HA and NA of the H5N1 AIV strains were analyzed by a NetNGlyc 1.0 Server http://www.cbs.dtu.dk/services/NetNGlyc/ (accessed on 12 October, 2022).

Furthermore, the nucleotide sequences were assembled and aligned using Geneious Prime 2022.2.2 (https://www.geneious.com (accessed on 12 October 2022)). Representative global and Egyptian avian influenza H5 sequences were retrieved from the public database (NCBI) and Global Initiative on Sharing All Influenza Data (GISAID) database. Phylogenetic analyses were conducted by employing a maximum likelihood methodology based on the Akaike criterion after selecting the best fit modes using IQ-tree software version 1.1.3 [50]. Trees were finally viewed and edited using FigTree v1.4.4 software (http://tree.bio.ed.ac.uk/software/figtree/, accessed on 5 November 2022) and Inkscape v1.1.

## 3. Results

### 3.1. Detection of H5N1 AIV in Wild Birds in Egypt

Among the 190 screened birds, few birds were apparently healthy while other birds exhibited neurological signs such as paresis, opisthotonos, and head tilt. In addition, greater-flamingo (*Phoenicopteridae*), true redstarts (*Phoenicurus*), and marbled-duck (*Marmaronetta angustirostris*) were found dead in a short time after capture without exhibiting any clinical symptoms (Table 1). All influenza positive birds had evidence of multifocal necrosis in the pancreas and liver as well as pulmonary congestion, edema, subepicardial bleeding, and myocarditis at the postmortem inspection (Table 1). The RT-PCR screening results were negative for NDV, IBV, ILT, IBDV, and WNV, but six samples were identified as positive for AIV of the H5N1 subtype (Table 1). Positive samples for the H5N1 subtype were found in three different Egyptian governorates (greater flamingo (*n* = 8) from Ismailia, common blackbird (*n* = 5), greater flamingo (*n* = 4), red-back-shrike (*n* = 4), marbled teal (*n* = 4) from Damietta, and ibis (*n* = 4) from Giza). None of the samples collected in the Beni-Suef governorate were positive. However, several true redstarts (*Phoenicurus*) were found dead, but the samples were found to be negative for all of the tested viruses. Six positive H5N1 samples were successfully isolated in embryonated chicken eggs and showed HA titers of 7–8 HA units. Furthermore, the obtained allantoic fluid was analyzed by RT-PCR and confirmed as H5N1.

### 3.2. Genetic Characterization Revealed Close Related Features to H5N1 Viruses Circulating in Different Countries

The blast search of the HA and NA gene segments confirmed that the AIV strains identified in this study belonged to the H5N1 subtype of clade 2.3.4.4b. The amino acid and nucleotide identities of the HA genes were 99–100% and 98–99%, respectively, to the H5N1 strains found in Nigeria and Lesotho in 2021. The HA showed a 97–99% nucleotide identity to the HA of the Egyptian HPAI H5N8 strains (clade 2.3.4.4b) reported between 2018 and 2021, but only 89–91% nucleotide identity to the HA of the Egyptian HPAI H5N1 strain (clade 2.2.1.2). The NA showed similar nucleotide identities with the H5N1 strains reported in Nigeria (97–99%) and Lesotho (98–99%) in 2021, while only 89–90% identity with the Egyptian H5N1-NA of clade 2.2.1.2 Other gene segments (PB2, PB1, PA, NP, M, and NS) showed nucleotide similarities between 99.03 and 99.67% with contemporary H5N1 strains from Nigeria and Bangladesh.

The HA genes in this study harbor multiple basic amino acids at their cleavage site PLREKRRKR/GLF (Table 2), similar to H5N1 of clade 2.3.4.4 found in West Africa and Europe in 2021–2022, demonstrating a highly pathogenic form [51,52]. The receptor binding sites of the HA protein of Egyptian H5N1 in this study revealed amino acids Q222, and G224 (H5 numbering), suggesting an avian-like α2,3-sialic acid receptor binding preference [53,54]. Similar to virus strains from West Africa and Europe, seven N-glycosylation sites were detected in the HA coding protein at amino acid positions 26, 27, 39, 181, 302, 500, and 559; however, N-glycosylation at amino acid position 500 was not found in the A/Greater-Flamingo/Egypt/31/2022 H5N1 virus.

The NA sequences of the Egyptian H5N1 strains in this study were found without amino acid deletion in the NA stalk region, similar to the H5N1 strains reported from Europe 2021–2022, but unlike the H5N1 strains identified in Nigeria and Benin in 2021. The NA coding genes in all of the sequenced isolates showed no substitutional amino acid mutation related to oseltamivir antiviral resistance and harbored 119E, 199D, 223I, 275H, and 293R. Furthermore, seven glycosylation sites were found within the NA coding protein at positions 50, 58, 63, 68, 88, 146, and 235. Similar glycosylation sites were found in the HPAI H5N1 strains reported from Lesotho, Nigeria, and the Czech Republic.

Furthermore, a few amino acid mutations that are associated with adaptation to poultry or increased replication in the mammalian hosts were found in the whole genome sequenced isolate (Table 2). The A/Ibis/Egypt/RLQP/229S/2022 exhibited amino acids associated with avian viral preference as 627E and 701D of PB2 (Table 2). The absence of substitutional mutations at amino acid positions 26, 30, 31, and 34 within the M2 sequence indicated no genetic markers related to the resistance to amantadine. The NS1 PDZ motif ESEV (227-230) was found in the Egyptian H5N1 isolate, which is typically shown in HPAI-H5N1, with no deletion in the NS1 of the strain under study (Table 2), as previously reported in the West African H5N1 strains of clade 2.3.4.4 [21]. However, the A/Ibis/Egypt/RLQP/229S/2022 isolate carries mammalian adaption and virulence traits such as PB2 (504V), PB1 (13P and 598L), PB1-F2 (82S), PA (127V, 672L, and 550L), NP (398Q), M2 (64S), and NS1 (42S). Similar amino acid features have been found in HPAI H5N1 strains of clade 2.3.4.4b reported from Africa and Europe.

### 3.3. Phylogenetic Relatedness with Viruses from Clade 2.3.4.4b

**Figure 1:**
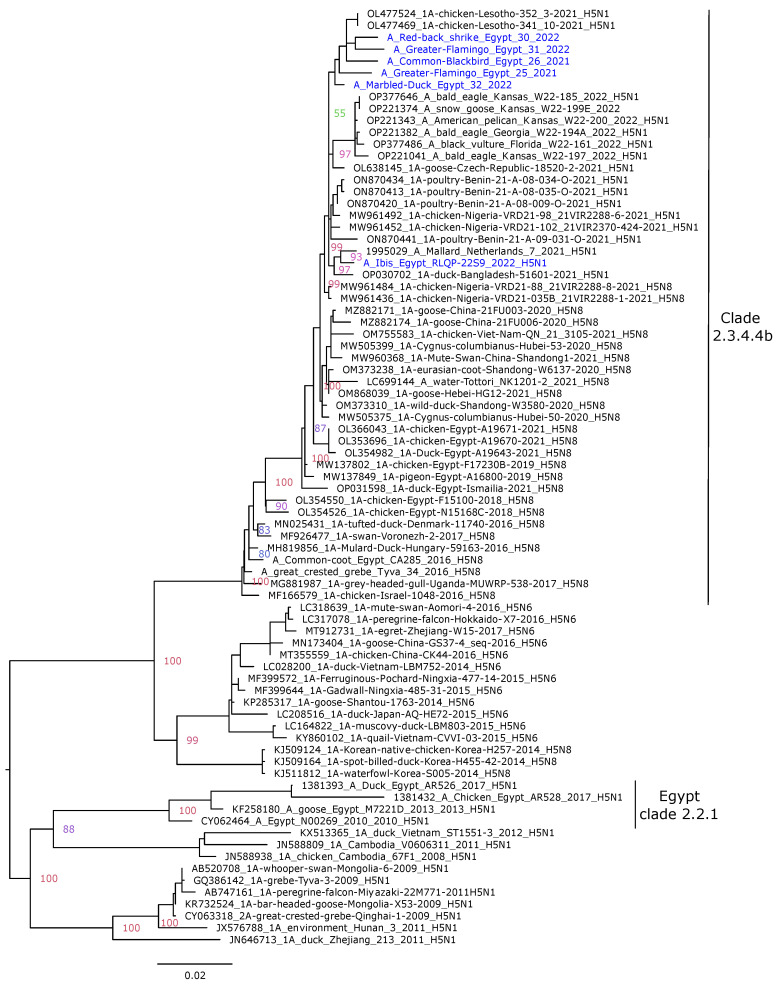
Phylogenetic trees of the nucleotide sequences of the HA gene segments of the avian influenza H5 subtype. Maximum likelihood calculations were performed using the IQTree software under the best fit model according to the Bayesian criterion (GTR+F+G4). Egyptian HPAI H5N1 viruses are colored in blue.

**Figure 2:**
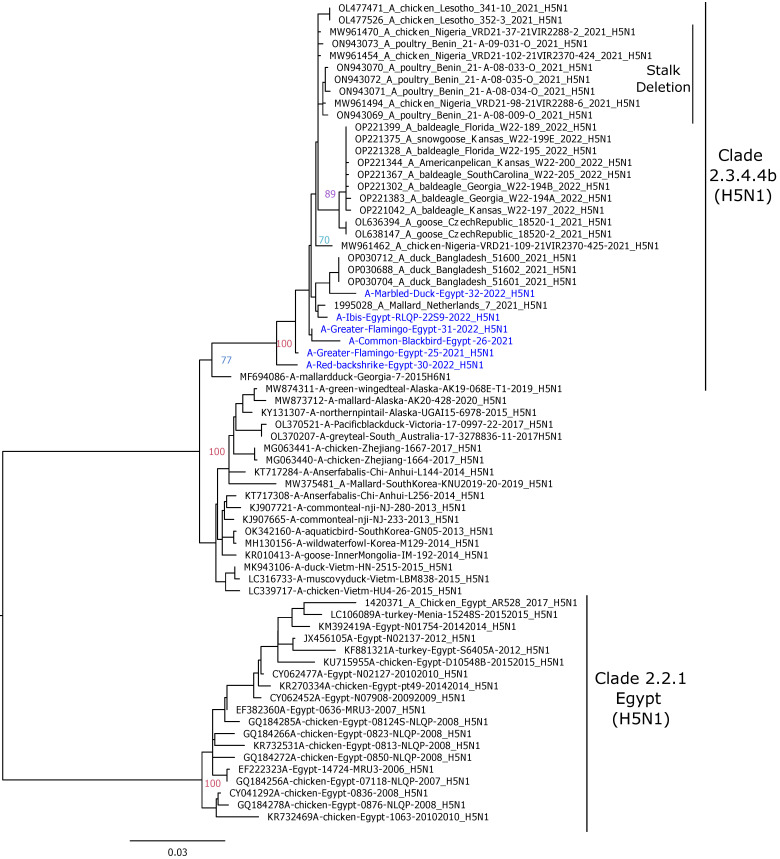
Phylogenetic trees of the nucleotide sequences of the NA gene segments of the avian influenza H5 subtype. Maximum likelihood calculations were performed using the IQTree software under the best fit model according to the Bayesian criterion. Egyptian HPAI H5N1 viruses are colored in blue.

To reveal the phylogenetic features of the Egyptian HPAI H5N1 strains isolated in the current study, phylogenetic analyses were performed using representative AIV strains. These analyses were found to corroborate the results of the genetic findings and revealed that the Egyptian HPAI H5N1 strains were closely related to the H5N1 strains circulating in Europe, Asia, and Africa during 2021–2022. The HA gene segments of the A/Ibis/Egypt/RLQP/229S/2022 Egyptian HPAI H5N1 isolate were phylogenetically closely related to strains detected in Bangladesh, Nigeria, and Europe (The Czech Republic and the Netherlands) of clade 2.3.4.4b (Figure 1). The HAs of the remaining five isolates were clustered more closely, related to H5N1 variants from Lesotho. The H5N1 strains of clade 2.3.4.4b; with a 20 amino acid deletion in the stalk region of the NA coding gene (Nigeria and Benin) were clustered phylogenetically from other strains with no deletions (Figure 2). The NAs of the Egyptian H5N1 strains in this study were close to the H5N1 strains circulating in Europe (The Netherlands)/Africa (Nigeria)/Asia (Bangladesh) within group 2.3.4.4b, showing no 20 amino acid deletions. Phylogenetic analyses of the PB2, PB1, PA, NP, M, and NS gene segments revealed different clustering, suggesting evidence of several reassortments within the H5N1 strains of clade 2.3.4.4b, as described previously [24]. The internal gene segments of the A/Ibis/Egypt/RLQP/229S/2022 virus revealed a close phylogenetic relation with the H5N1 strains (clade 2.3.4.4b) isolated in Africa in 2021–2022 (Supplementary Figure S1a–f).

## 4. Discussion

Wild migratory birds serve as natural reservoirs for AIV and the majority of the variants are low-pathogenic subtypes [55]. Since late 2020, HPAI H5N1 variants of clade 2.3.4.4b have become the dominant highly pathogenetic subtype detected in both wild and domestic birds [24]. This massive global dissemination of the HPAI H5N1 variants of clade 2.3.4.4b have caused a severe negative impact on both wildlife and the poultry industry [24] as well as a threat to humans [25,26]. Wild migratory birds have been linked to the introduction of different AIV subtypes into Egypt [11]. In this study, the HPAI H5N1 subtype of clade 2.3.4.4b from Egypt in 2021–2022 was isolated and genetically characterized.

Recent studies have indicated that clade 2.3.4.4b H5N1 became predominant over 2.3.4.4b H5N8, with high prevalence and spread in wild birds and further transmission to domestic poultry in various countries [19]. Here, virus strains isolated in this study were characterized as HPAI H5N1 strains, and are genetically and phylogenetically closely related to H5N1 strains of clade 2.3.4.4b circulating in Europe, Asia, and Africa during 2021–2022. The high genetic and phylogenetic relatedness between the strains in this study and other HPAI H5N1 strains of clade 2.3.4.4b, particularly from Nigeria, Bangladesh, and the Netherlands, suggests that the Egyptian H5N1 strains followed the same way as HPAI H5N1 introduction in those countries via wild birds, possibly via the Black Sea/Mediterranean migratory bird flyway. In particular, this demonstrates the role of wild migratory birds in the spread and continuous incursions of influenza A virus subtypes over the years and among countries. In general, the wild birds sampled in this study, exhibiting neurological symptoms, were found to be positive for HPAI with a few dead birds. Samples collected from True redstarts were found to be negative for AIV, NDV, IBV, ILT, IBDV, and WNV. Furthermore, amino acid analyses of HA and other internal proteins revealed the presence of many substitutions related to virulence in birds as well as other amino acids previously reported in association with mammalian preference. Natural replacement among circulating AIV subtypes was observed in Egypt after the introduction of HPAI H5N8 in late 2016, where it replaced HPAI H5N1 variants of clade 2.2.1.2 [39]. Fortunately, vaccination was implemented at that time as a control strategy in the poultry industry and could be updated to include an H5N1 clade 2.3.4.4b-based vaccine [56,57]. It might now be a similar ongoing replacement, which requires further studies including a cross antigenic test with antisera retrieved from different H5 vaccines in Egypt and potentially updating the AIV vaccination program for Egypt. Moreover, the presence of bacteria or other viruses cannot be excluded, which calls for the further deep sequencing (metagenomics) of such samples.

Taken together, variants of HPAI H5N1 of clade 2.3.4.4b have continued to circulate in wild birds around the globe, resulting in an introduction to different countries. The detection of the HPAI H5N1 strains in wild birds in Egypt during 2021–2022, with close genetic and phylogenetic relatedness to H5N1 reported recently in Europe and Africa, highlights the probability of virus transmission to different poultry sectors in Egypt. Therefore, continuous surveillance of emerging AIVs in close proximity to the wild–domestic bird interface is essential for earlier virus detection and preemptive intervention before the establishment of the virus in poultry populations in Egypt and its possible threat to human health.

## Figures and Tables

**Table 1:** Epidemiological data, symptoms of birds, and RT-PCR results in a study on viruses in migratory birds in Egypt.

Collection Date	Sampling Governorate	Type of Birds		Number of Birds	Clinical Signs	Post Mortem Examination	No. of Positive Samples	qRT-PCR (Ct)
		Common Name	Scientific Name					
10/2021	Ismailia (Berket el Baalwa)	Marbled Teal	Marmaronetta angustirostris	7	Lethargy and inability to fly	−	−	−
		Red-back-shrike	Lanius collurio	15	Neurological impairment	−	−	−
		Greater-Flamingo	Phoenicopteridae	8	Dead	+	1	AIV (31)
		Common Snipe	Gallinago gallinago	7	Lethargy and inability to fly	−	−	−
		Tufted Duck	Aythya fuligula	5	Lethargy and inability to fly	−	−	−
11/2021	Damietta (Ras El-bar)	Common-Blackbird	Turdus merula	5	Neurological impairment	++	1	AIV (25)
		Eurasian Jay	Garrulus glandarius	22	Apparent healthy	−	−	−
		True redstarts	Phoenicurus	18	Lethargy and inability to fly	−	−	−
		Wrynecks	Jynx	30	Lethargy and inability to fly	−	−	−
1/2022	Giza	Dusky Warbler	Phylloscopus fuscatus	5	Lethargy and inability to fly	−	−	−
		Squacco Heron	Ardeola ralloides	7	Lethargy and inability to fly	−	−	−
		Ibis	Threskiornithinae	4	Neurological impairment	++	1	AIV (25)
	Beni-Suef	Curlews	Numenius	2	Lethargy and inability to fly	−	−	−
		Gadwall	Anas strepera	3	Lethargy and inability to fly	−	−	−
		Caspian Plover	Charadrius asiaticus	4	Lethargy and inability to fly	−	−	−
3/2022	Damietta (Lake Manzala)	Spotted Sandgrouse	Pterocles senegallus	4	Lethargy and inability to fly	−	−	−
		Greater-Flamingo	Phoenicopteridae	4	Neurological impairment	++	1	AIV (32)
		True redstarts	Phoenicurus	6	Dead	−	−	−
		Woodchat Shrike	Lanius senator	7	Lethargy and inability to fly	−	−	−
		European Bee-eater	Merops apiaster	5	Lethargy and inability to fly	−	−	−
		Red-back-shrike	Lanius collurio	4	neurological impairment	++	1	AIV (28)
		Common-Blackbird	Turdus merula	15	Lethargy and inability to fly	−	−	−
		Marbled Duck	Marmaronetta angustirostris	3	Dead	+	1	AIV (29)

**−** = no post mortem finding or no amplification to any of the tested viruses under study (H5N1, H9N2, H5N8, H6N2, NDV, IBV, ILT, IBDV, and West Nile fever virus). + = multifocal necrosis in the pancreas and liver as well as pulmonary congestion and edema, subepicardial bleeding, and myocarditis. ++ = general septicemia and brain congestion. Underlined = bird species that showed positive results for avian influenza virus (AIV).

**Table 2:** Key amino acid residue analysis of the Egyptian HPAI H5N1 viruses among different proteins.

	HA							NA Stalk Del	PB2		PB1-F2 Length	M “Amantadine Resistance Markers”					NS1 Length
	Receptor Binding Sites						Cleavage Site		627	701							
	103	129	186	221	222	224						26	27	30	31	34	
A/Duck/Jiangsu/k1203/2010 ^1^	H	L	E	G	Q	G	PLREKRRKRGLF	No	E	D	90	L	V	A	N	G	225
A/duck/Zhejiang/6D18/2013 ^2^	H	L	E	G	Q	G	PLREKRRKRGLF	No	E	D	52	L	V	A	N	G	230
A/turkey/Germany/AR3390-L00939/2014 ^3^	H	L	E	G	Q	G	PLRERRRKRGLF	No	E	D	52	L	V	A	N	G	237
A/great_crested_grebe/Uvs-Nuur_Lake/341/2016 ^4^	H	L	E	G	Q	G	PLREKRRKRGLF	No	E	D	52	L	V	A	S	G	230
A/ A/common-coot/Egypt/CA285/2016 ^5^	H	L	E	G	Q	G	PLREKRRKRGLF	No	E	D	52	L	V	A	S	G	217
A/Chicken/Egypt/AR528/2017 ^6^	H	x	A	G	Q	G	PQGEKRRKKRGLF	present	K	D	90	L	V	A	S	G	230
A/chicken/Lesotho/352.3/2021^7^	H	L	E	G	Q	G	PLREKRRKRGLF	No	E	D	90	L	V	A	S	G	230
A/chicken/Nigeria/VRD21-98/21VIR2288/2021 ^8^	H	L	E	G	Q	G	PLREKRRKRGLF	present	E	D	90	L	V	A	S	G	230
A/goose/CzechRepublic/18520/1/2021 ^9^	H	L	E	G	Q	G	PLREKRRKRGLF	No	E	D	90	L	V	A	S	G	230
A/Greater-Flamingo/Egypt/25/2021 #	H	L	E	G	Q	G	PLREKRRKRGLF	No	-	-	-	-	-	-	-	-	-
A/Common-Blackbird/Egypt/26/2021 #	H	L	E	G	Q	G	PLREKRRKRGLF	No	-	-	-	-	-	-	-	-	-
A/Red-back-shrike/Egypt/30/2022 #	H	L	E	G	Q	G	PLREKRRKRGLF	No	-	-	-	-	-	-	-	-	-
A/Greater-Flamingo/Egypt/31/2022 #	H	L	E	G	Q	G	PLREKRRKRGLF	No	-	-	-	-	-	-	-	-	-
A/Marbled-Duck/Egypt/32/2022 #	H	L	E	G	Q	G	PLREKRRKRGLF	No	-	-	-	-	-	-	-	-	-
A/Ibis/Egypt/RLQP/229S/2022 *	H	L	E	G	Q	G	PLREKRRKRGLF	No	E	D	90	L	V	A	S	G	230

1 = First isolated H5N8 in China; 2 = H5N8 reference strain of clade 2.3.4.4a in Asia. 3 = H5N8 reference strain of clade 2.3.4.4a in Europe; 4 = H5N8 reference strain of clade 2.3.4.4b in Asia. 5 = First reported H5N8 virus in Egypt; 6 = Representative H5N1 clade 2.2.1.1 in Egypt. 7 = Representative H5N1 clade 2.3.4.4b in Lesotho; 8 = Representative H5N1 clade 2.3.4.4b in Nigeria. 9 = Representative H5N1clade 2.3.4.4b in Europe; x = not found (deletion). - = not analyzed; # = Egyptian virus strains, for which the HA and NA sequences were obtained in this study. * = Egyptian virus which whole genome sequence is obtained in this study.

## Data Availability

Nucleotide sequence generated in this study was uploaded at the GenBank NCBI and the accession numbers are provided in the Supplementary Table S3.

## Supplementary Materials

The following supporting information can be downloaded at: https://www.mdpi.com/article/10.3390/pathogens12010090/s1, Figure S1: Phylogenetic analyses of the PB2(a), PB1(b), PA(c), NP(d), M(e), and NS(f) gene segments.; Table S1: Primers and probes used for RT-PCR identification of viruses in collected samples.; Table S2: Primers used in Reverse Transcriptase-Polymerase Chain Reaction (RT-PCR) for sequencing.; Table S3: Epidemiological data and GenBank accession number of detected avian influenza viruses [58,59].
