# Supplementary material for: Emergence of Highly Pathogenic Avian Influenza A Virus (H5N1) of Clade 2.3.4.4b in Egypt, 2021–2022
Source: Pathogens. 2023 Jan 5;12(1):90. doi: 10.3390/pathogens12010090 (PMC9863303; doi:10.3390/pathogens12010090)
Supplement: Supplementary file 1 [file pathogens-12-00090-s001.zip › pathogens-2115535-supplementary.pdf]

Supplementary tables and figures for manuscript:

Emergency and genetic characterization of Highly Pathogenic influenza A(H5N1) virus of clade 2.3.4.4 in Egypt, 2021-2022

Supplementary table S1. Primers and probes used for RT-PCR identification of viruses in collected samples.

ID	Primer sequences	Reference
**AIV-M-gene**	sep1: AGATGAGTCTTCTAACCGAGGTCG<br>sep2: TGCAAAAACATCTTCAAGTCTCTG<br>sep-probe: FAM-TCAGGCCCCCTCAAAGCCGA-TAMRA	[1]
**AIV-H5 subtype**	H5LH1: ACATATGACTACCCACARTATTCAG<br>H5RH1: AGACCAGCTAYCATGATTGC<br>H5PRO: FAM-TCWACAGTGGCGAGTTCCCTAGCA-TAMRA	[2]
**AIV-H6 subtype**	IAV-H6-1666F: CTTGGTGTGTATCAAATYCTTGC<br>IAV-H6-1776R: CATTGARCCATTTGARCACATCCA<br>IAV-H6-1693: FAM-TATAGTACGGTATCGAGCAGYCT-MGB	[3]
**AIV-H9 subtype**	For: GGAAGAATTAATTATTATTGGTCGGTAC<br>Rev: GCCACCTTTTTCAGTCTGACATT<br>H9 probe: FAM-AACCAGGCCAGACATTGCGAGTAAGATCC-TAMRA	[4]
**AIV-N1 subtype**	N1 forward: TAYAACTCAAGGTTTGAGTCTGTYGCTTG<br>N1 reverse: ATGTTRTTCCTCCAACTCTTGATRGTGTC<br>N1-Probe: FAM-TCAGCRAGTGCYTGCCATGATGGCA-TAMRA	[3]
**AIV-N2 subtype**	FN2: TGGACAGGGAACAACACTAAA C<br>RN2: ACAAGCCTCCCATCGTAAAT<br>N2-Probe: TXRED-CAAATGAAATGGAACACCCAACTCAT-BHQ23	[3]
**AIV-N8 subtype**	N8-1296F: TCCATGYTTTGGGTTGARATGAT<br>N8 1423R: GCTCCATCRTGCCAYGACCA<br>N8 A354-Probe: FAM-TCHAGYAGCTCCATTGTRATGTGTGGAGT-TAMRA	[3]
**IBV**	AIBV-fr: ATGCTCAACCTTGTCCCTAGCA<br>AIBV-as: TCAAACTGCGGATCATCACGT<br>AIBV-TM: FAM-TTGGAAGTAGAGTGACGCCCAAACTTCA-TAMRA	[5]
**NDV**	F+4839: TCCGGAGGATACAAGGGTCT<br>F-4939: AGCTGTTGCAACCCCAAG<br>F+4894: FAM-AAGCGTTTCTGTCTCCTTCCTCCA-TAMRA	[6]
**ILTV**	ILTVgCU771: CCTTGCGTTTGAATTTTTCTGT<br>ILTVgCL873: TTCGTGGGTTAGAGGTCTGT<br>ILTVprobe817: FAM-CAGCTCGGTGACCCCATTCTA-MGBNFQ	[7]

**WNV**	WNV10668: CAGACCACGCTACGGCG<br>WNV11770: CTAGGGCCGCGTGGG<br>WNV10692: FAM-TCTGCGGAGAGTGCAGTCTGCGAT-TAMRA	[8]
**IBDV**	F/AUS GU: TCACCG TCCTCAGCTTACCCACATC<br>R/AUS GL: GGATTTGGGATCAGCTCGAAGTTGC	[9]

Supplementary table S2. Primers used in Reverse Transcriptase-Polymerase Chain Reaction (RT-PCR) for sequencing.

FLI= Friedrich-Loeffler-Institut; NLQP= The National laboratory for quality control on poultry production; SEPRL= The Southeast Poultry Research Laboratory.

Primer ID	Nucleotide Sequence	Reference
	**PB2 Primers**	
**PB2-1FV2**	5'GGAAAGCAGGTCAAATATATTCAATA 3'	**FLI**
**PB2-F1361**	5’ATTGATAATGTCATGGGGATG 3’	**NLQP**
**PB2-1421R**	5’ GGT AAT AAT CCG ATC ATC CCC AT 3’	**NLQP**
**PB2-F-1775**	5'TAG TAC CAA AGG CTG CCA GA 3'	**NLQP**
**PB2-1RV2**	5'GGTCGTTTTTAAACAATTCGACAC 3'	**FLI**
**PB2-R828**	5'TCTCCAGCAGTGATGCCAGTG 3'	**NLQP**
	**PB1 Primers**	
**PB1-2FV3**	5’AGCAGGCAAACCATTTGAATGG3’	**FLI**
**PB1-2F -1100**	5’AC ACA AAT ACC AGC AGA AAT GCT 3’	**NLQP**
**PB1-F-1433**	5’GCA AGC TAG TCG GAA TCA A 3’	**NLQP**
**Pb1 R-867**	5’CAC GAC GTT TGC CAA TTT AGC C 3’	**NLQP**
**PB1-1230R**	5’ATGTTGAACATGCCCATCATCAT3’	**NLQP**
**PB1-R-2300**	5’TTC ATG AAG GAC AAG CTA A 3’	**NLQP**
	**PA Primers**	
**PA -3FV2**	5'AGC GAA AGC AGG TAC TGA TTC AAA A 3'	**FLI**
**PA+734**	5'AACCGAACGGCTGCATTGA 3'	**NLQP**
**PA-1131F**	5'ACT CGG TGA GAA CAT GGC ACC3'	**NLQP**
**PA-F1825**	5'ACATGACCAAAGAATTCTTTGA3'	**NLQP**
**PA -1312R**	5'CTC CTA TTT CAT CAA GTT CTA TCC 3'	**NLQP**
**PA-R-1621**	5'CTT TTC CCA CTT GTG TGG CTC C3'	**NLQP**
**PA-R-2193**	5'ATA GTA GCA TTG CCA CAA CTA 3'	**NLQP**
	**HA Primers**	
**Bm-NS-890R**	**-ATA TCG TCT CGT ATT AGT AGG AAA CAA GGG TGT TTT**	[10,11]
**Bm-HA-1-**	**TAT TCG TCT CAG GGA GCA AAA GCA GGG G**	
**KH3-**	**TAC CAA CCG TCT ACC ATK CCYTG**	
**KH1-**	**CCTCCAGARTATGCMTAYAAAATTGTC**	
**4FV2**	**AGCAGGGGTTCAATCTGTCAAAA**	
**4RV2**	**AGTAGAAACAAGGGTGTTTTTAACTA**	
	**NP Primers**	

**NP-5FV2**	5'CA CCG AGT GAC ATC AAC ATC A3'	**FLI**
**NP-F370**	5'GAT AAT CAC TCA CCG AGT GAC ATC3'	**NLQP**
**NP-5RV2**	5'AAG GGT ATT TTT CTT TAA TTG TCA T A C3'	**FLI**
**NP-R764**	5'TCC ATC ATT GCT CTT TGT3'	**NLQP**
**NA Primers**		
**6FV2**	**AGCAAAAGCAGGAGTTTAAAATGA**	[10,11]
**BA-NA-1**	**TAT TGG TCT CAG GGA GCA AAA GCA GGA GT**	
**N1- 608-F**	**AAT AAC AGA CAC CAT CAA GAG TTG**	
**BaNA-1413-R-**	**ATA TGG TCT CGT ATT AGT AGA AAC AAG GAG TTT TTT**	
**N1-R-720 -**	**CAT GCA CAT TCA GAC TCT TG**	
**6RV2-**	**TAGAAACAAGGAGTTTTTTGAACAAAC**	
**M Primers**		
**M-7FV2**	**5'**CAAAAGCAGGTAGATGTTGAAAGA **3'**	**FLI**
**M-7RV2**	5'AACAAGGTAGTTTTTTACTCCAATTC 3'	**FLI**
**M-F710**	5'GTTCTTGAAAATTTGCAGG C 3'	**NLQP**
**M-R424**	5'GTTGTATGAGACCCATGC 3'	**NLQP**
**NS Primers**		
**NS-8FV2**	**5'**AAA AGC AGG GTG ACA AAG ACA TAA **3'**	**FLI**
**NS-8RV2**	**5'**AGT AGA AAC AAG GGT GTT TTT TAT CA **3'**	**FLI**
**NS-R598**	**5'**GAG TTA TCA TTC CAT TCA AGT CC **3'**	**SEPRL**
**NS-F327**	**5'**GACTGG TTC ATG CTC ATG CC **3'**	**SEPRL**

Table S3. Epidemiological data and GenBank accession number of detected avian influenza viruses

	**Strain name**	**Collection date**	**Location**	**Genbank Accession number**
1	A/Greater-Flamingo/Egypt/25/2021	October 2021	Ismailia	OP413044; OP413049
2	A/Common-Blackbird/Egypt/26/2021	November 2021	Damietta	OP413045; OP413050
3	A-Ibis-Egypt-RLQP-229S-2022-H5N1	January 2022	Giza	OP491851; OP491852; OP491854 ;OP491855; OP491860; OP491856; OP491857; OP491859
4	A/Red-back-shrike/Egypt/30/2022	March 2022	Damietta	OP413046; OP413051
5	A/Greater-Flamingo/Egypt/31/2022	March 2022	Damietta	OP413047; OP413052
6	A/Marbled-Duck/Egypt/32/2022	March 2022	Damietta	OP413048; OP413053

Supplementary Figure S1 Phylogenetic analyses of the PB2(a), PB1(b), PA(c), NP(d), M(e), and NS(f) gene segments.
Phylogenetic trees include representative strains from recent H5N1 subtypes from both Eurasian and North American lineages; in addition to representative H5N1 (clade 2.2.1.2) strains from Egypt and other influenza virus subtypes showing a high nucleotide similarity. The Egyptian HPAI H5N1 strain sequenced in this study is colored in blue. Bootstrap values are shown on main nodes in the phylogenetic tree

a-PB2

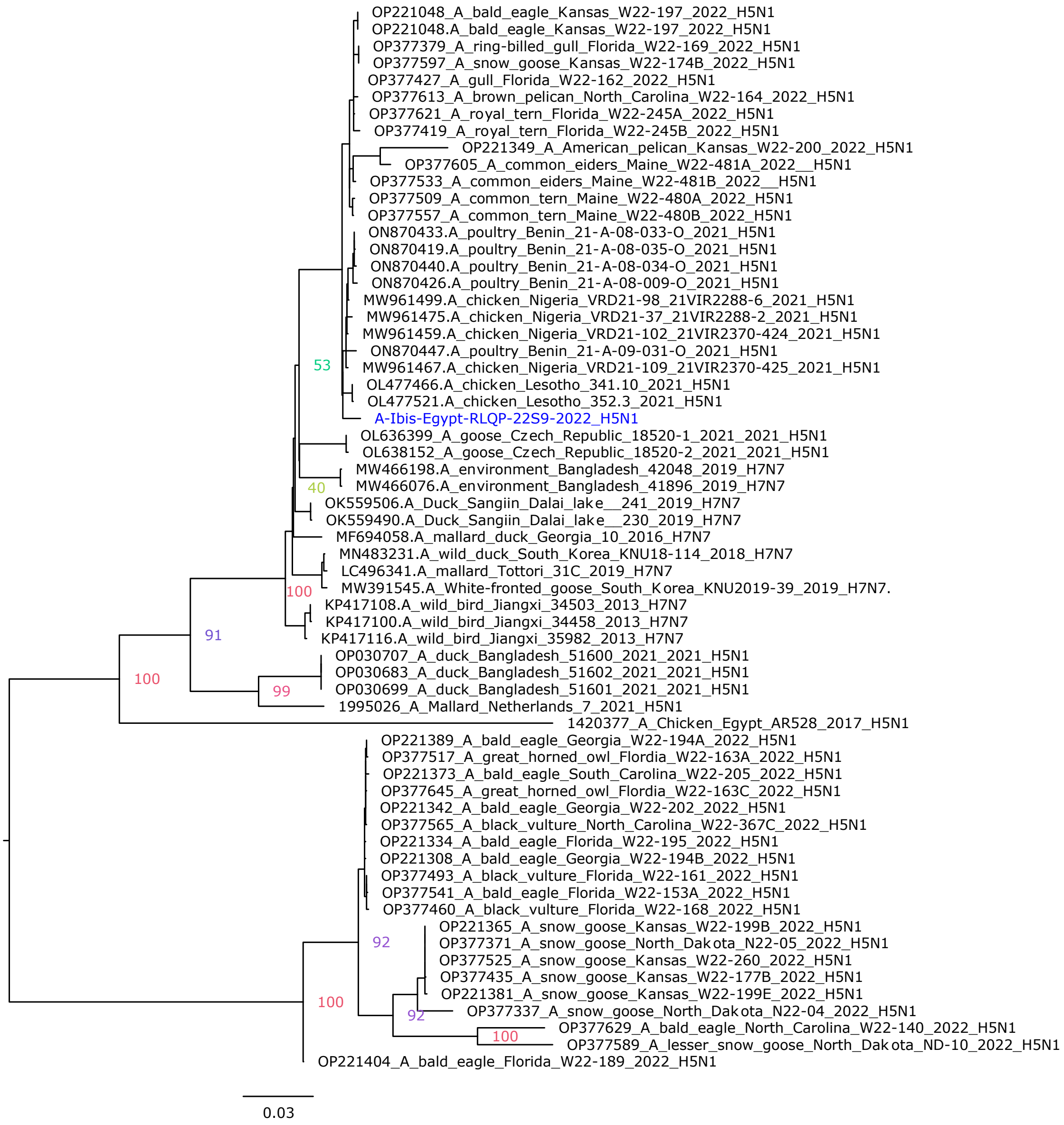

b-PB1

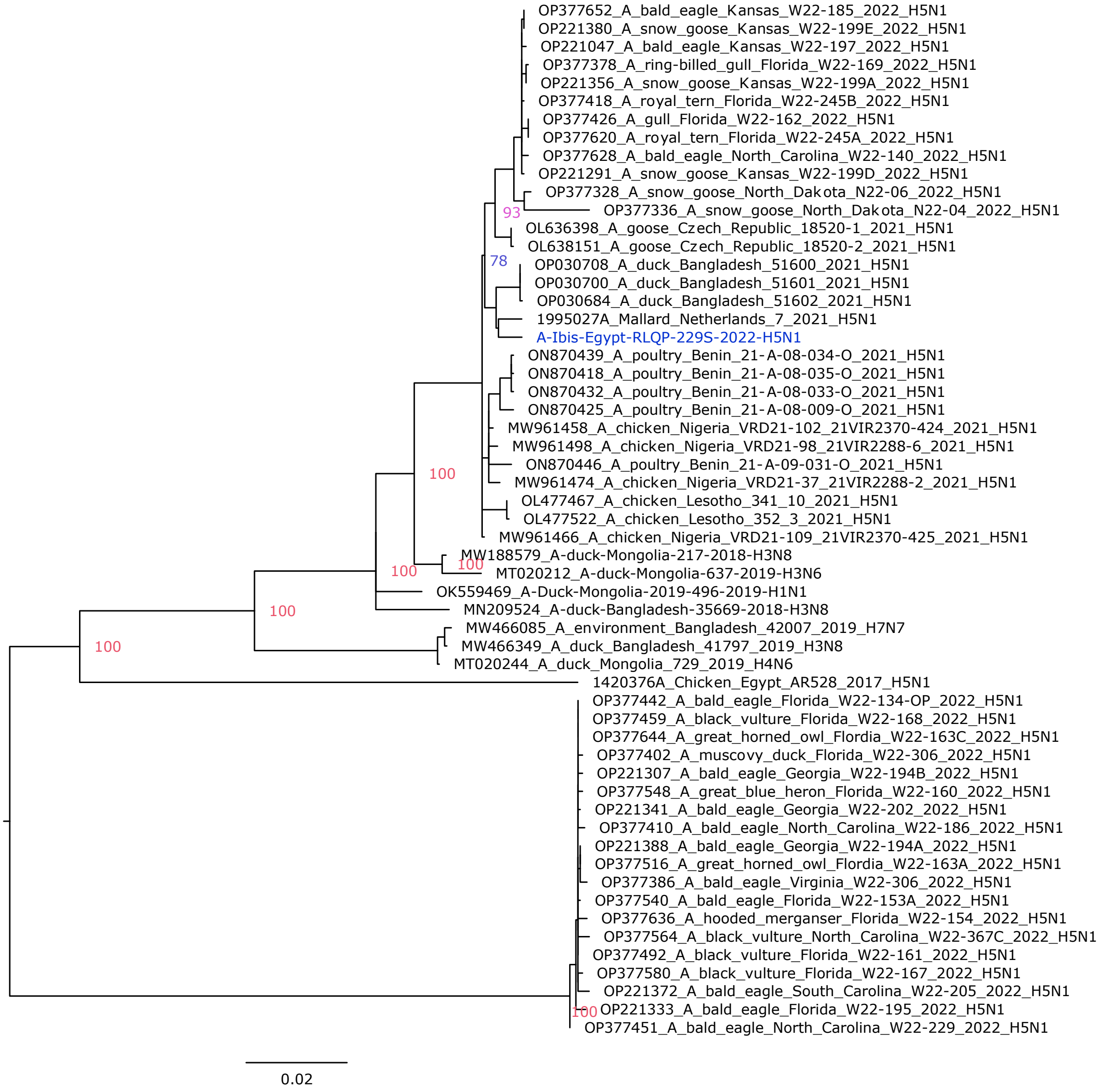

OP377652_A_bald_eagle_Kansas_W22-185_2022_H5N1
OP221380_A_snow_goose_Kansas_W22-199E_2022_H5N1
OP221047_A_bald_eagle_Kansas_W22-197_2022_H5N1
OP377378_A_ring-billed_gull_Florida_W22-169_2022_H5N1
OP221356_A_snow_goose_Kansas_W22-199A_2022_H5N1
OP377418_A_royal_tern_Florida_W22-245B_2022_H5N1
OP377426_A_gull_Florida_W22-162_2022_H5N1
OP377620_A_royal_tern_Florida_W22-245A_2022_H5N1
OP377628_A_bald_eagle_North_Carolina_W22-140_2022_H5N1
OP221291_A_snow_goose_Kansas_W22-199D_2022_H5N1
OP377328_A_snow_goose_North_Dakota_N22-06_2022_H5N1
93
OP377336_A_snow_goose_North_Dakota_N22-04_2022_H5N1
OL636398_A_goose_Czech_Republic_18520-1_2021_H5N1
OL638151_A_goose_Czech_Republic_18520-2_2021_H5N1
78
OP030708_A_duck_Bangladesh_51600_2021_H5N1
OP030700_A_duck_Bangladesh_51601_2021_H5N1
OP030684_A_duck_Bangladesh_51602_2021_H5N1
1995027A_Mallard_Netherlands_7_2021_H5N1
A-Ibis-Egypt-RLQP-229S-2022-H5N1
ON870439_A_poultry_Benin_21-A-08-034-O_2021_H5N1
ON870418_A_poultry_Benin_21-A-08-035-O_2021_H5N1
ON870432_A_poultry_Benin_21-A-08-033-O_2021_H5N1
ON870425_A_poultry_Benin_21-A-08-009-O_2021_H5N1
MW961458_A_chicken_Nigeria_VRD21-102_21VIR2370-424_2021_H5N1
MW961498_A_chicken_Nigeria_VRD21-98_21VIR2288-6_2021_H5N1
ON870446_A_poultry_Benin_21-A-09-031-O_2021_H5N1
100
MW961474_A_chicken_Nigeria_VRD21-37_21VIR2288-2_2021_H5N1
OL477467_A_chicken_Lesotho_341_10_2021_H5N1
OL477522_A_chicken_Lesotho_352_3_2021_H5N1
MW961466_A_chicken_Nigeria_VRD21-109_21VIR2370-425_2021_H5N1
MW188579_A-duck-Mongolia-217-2018-H3N8
100
100
MT020212_A-duck-Mongolia-637-2019-H3N6
OK559469_A-Duck-Mongolia-2019-496-2019-H1N1
100
MN209524_A-duck-Bangladesh-35669-2018-H3N8
MW466085_A_environment_Bangladesh_42007_2019_H7N7
100
MW466349_A_duck_Bangladesh_41797_2019_H3N8
MT020244_A_duck_Mongolia_729_2019_H4N6
1420376A_Chicken_Egypt_AR528_2017_H5N1
OP377442_A_bald_eagle_Florida_W22-134-OP_2022_H5N1
OP377459_A_black_vulture_Florida_W22-168_2022_H5N1
OP377644_A_great_horned_owl_Flordia_W22-163C_2022_H5N1
OP377402_A_muscovy_duck_Florida_W22-306_2022_H5N1
OP221307_A_bald_eagle_Georgia_W22-194B_2022_H5N1
OP377548_A_great_blue_heron_Florida_W22-160_2022_H5N1
OP221341_A_bald_eagle_Georgia_W22-202_2022_H5N1
OP377410_A_bald_eagle_North_Carolina_W22-186_2022_H5N1
OP221388_A_bald_eagle_Georgia_W22-194A_2022_H5N1
OP377516_A_great_horned_owl_Flordia_W22-163A_2022_H5N1
OP377386_A_bald_eagle_Virginia_W22-306_2022_H5N1
OP377540_A_bald_eagle_Florida_W22-153A_2022_H5N1
OP377636_A_hooded_merganser_Florida_W22-154_2022_H5N1
OP377564_A_black_vulture_North_Carolina_W22-367C_2022_H5N1
OP377492_A_black_vulture_Florida_W22-161_2022_H5N1
OP377580_A_black_vulture_Florida_W22-167_2022_H5N1
OP221372_A_bald_eagle_South_Carolina_W22-205_2022_H5N1
100
OP221333_A_bald_eagle_Florida_W22-195_2022_H5N1
OP377451_A_bald_eagle_North_Carolina_W22-229_2022_H5N1
0.02

c-PA

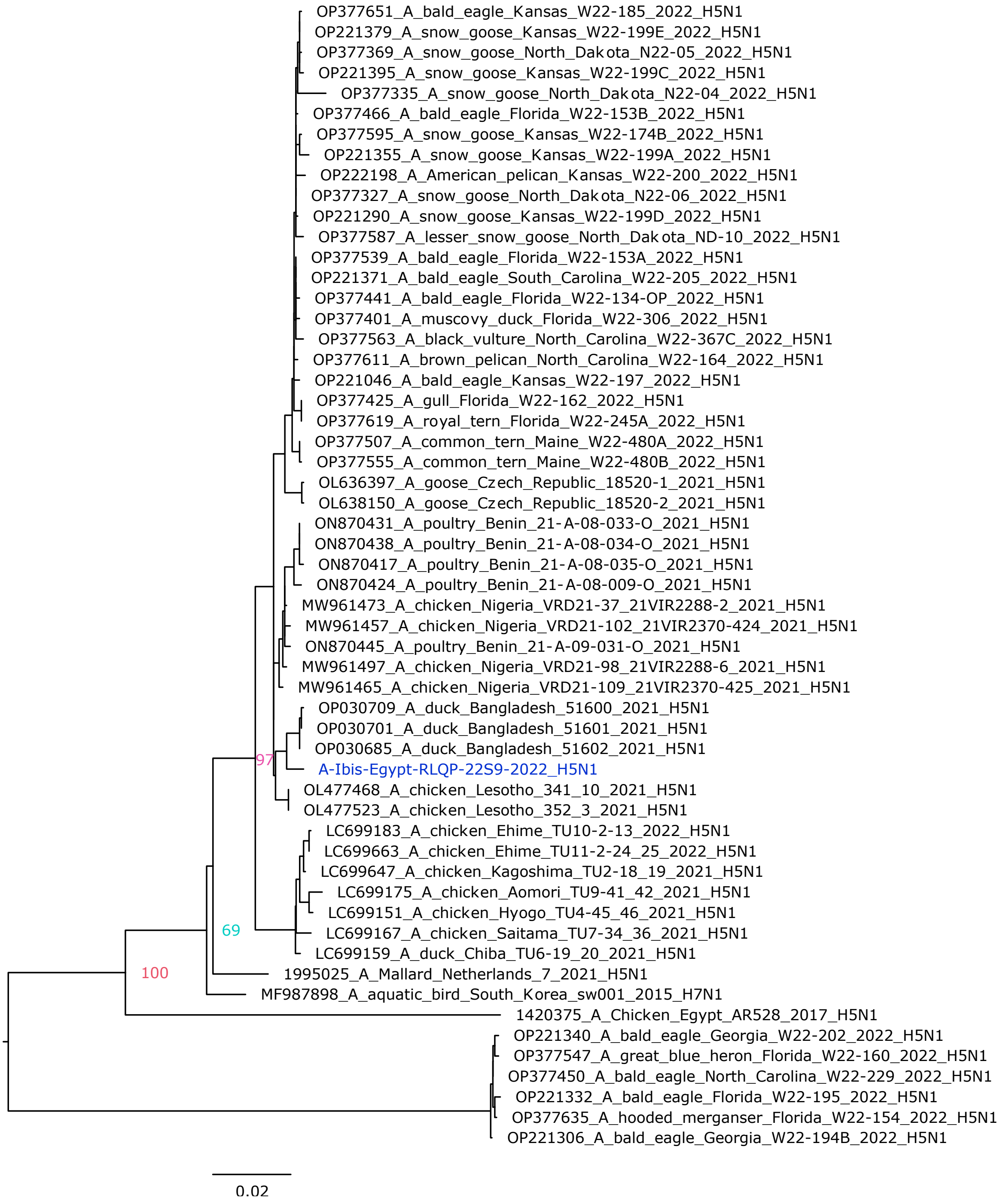
OP377651_A_bald_eagle_Kansas_W22-185_2022_H5N1
OP221379_A_snow_goose_Kansas_W22-199E_2022_H5N1
OP377369_A_snow_goose_North_Dakota_N22-05_2022_H5N1
OP221395_A_snow_goose_Kansas_W22-199C_2022_H5N1
OP377335_A_snow_goose_North_Dakota_N22-04_2022_H5N1
OP377466_A_bald_eagle_Florida_W22-153B_2022_H5N1
OP377595_A_snow_goose_Kansas_W22-174B_2022_H5N1
OP221355_A_snow_goose_Kansas_W22-199A_2022_H5N1
OP222198_A_American_pelican_Kansas_W22-200_2022_H5N1
OP377327_A_snow_goose_North_Dakota_N22-06_2022_H5N1
OP221290_A_snow_goose_Kansas_W22-199D_2022_H5N1
OP377587_A_lesser_snow_goose_North_Dakota_ND-10_2022_H5N1
OP377539_A_bald_eagle_Florida_W22-153A_2022_H5N1
OP221371_A_bald_eagle_South_Carolina_W22-205_2022_H5N1
OP377441_A_bald_eagle_Florida_W22-134-OP_2022_H5N1
OP377401_A_muscovy_duck_Florida_W22-306_2022_H5N1
OP377563_A_black_vulture_North_Carolina_W22-367C_2022_H5N1
OP377611_A_brown_pelican_North_Carolina_W22-164_2022_H5N1
OP221046_A_bald_eagle_Kansas_W22-197_2022_H5N1
OP377425_A_gull_Florida_W22-162_2022_H5N1
OP377619_A_royal_tern_Florida_W22-245A_2022_H5N1
OP377507_A_common_tern_Maine_W22-480A_2022_H5N1
OP377555_A_common_tern_Maine_W22-480B_2022_H5N1
OL636397_A_goose_Czech_Republic_18520-1_2021_H5N1
OL638150_A_goose_Czech_Republic_18520-2_2021_H5N1
ON870431_A_poultry_Benin_21-A-08-033-O_2021_H5N1
ON870438_A_poultry_Benin_21-A-08-034-O_2021_H5N1
ON870417_A_poultry_Benin_21-A-08-035-O_2021_H5N1
ON870424_A_poultry_Benin_21-A-08-009-O_2021_H5N1
MW961473_A_chicken_Nigeria_VRD21-37_21VIR2288-2_2021_H5N1
MW961457_A_chicken_Nigeria_VRD21-102_21VIR2370-424_2021_H5N1
ON870445_A_poultry_Benin_21-A-09-031-O_2021_H5N1
MW961497_A_chicken_Nigeria_VRD21-98_21VIR2288-6_2021_H5N1
MW961465_A_chicken_Nigeria_VRD21-109_21VIR2370-425_2021_H5N1
OP030709_A_duck_Bangladesh_51600_2021_H5N1
OP030701_A_duck_Bangladesh_51601_2021_H5N1
OP030685_A_duck_Bangladesh_51602_2021_H5N1
97
A-Ibis-Egypt-RLQP-22S9-2022_H5N1
OL477468_A_chicken_Lesotho_341_10_2021_H5N1
OL477523_A_chicken_Lesotho_352_3_2021_H5N1
LC699183_A_chicken_Ehime_TU10-2-13_2022_H5N1
LC699663_A_chicken_Ehime_TU11-2-24_25_2022_H5N1
LC699647_A_chicken_Kagoshima_TU2-18_19_2021_H5N1
LC699175_A_chicken_Aomori_TU9-41_42_2021_H5N1
LC699151_A_chicken_Hyogo_TU4-45_46_2021_H5N1
69
LC699167_A_chicken_Saitama_TU7-34_36_2021_H5N1
LC699159_A_duck_Chiba_TU6-19_20_2021_H5N1
100
1995025_A_Mallard_Netherlands_7_2021_H5N1
MF987898_A_aquatic_bird_South_Korea_sw001_2015_H7N1
1420375_A_Chicken_Egypt_AR528_2017_H5N1
OP221340_A_bald_eagle_Georgia_W22-202_2022_H5N1
OP377547_A_great_blue_heron_Florida_W22-160_2022_H5N1
OP377450_A_bald_eagle_North_Carolina_W22-229_2022_H5N1
OP221332_A_bald_eagle_Florida_W22-195_2022_H5N1
OP377635_A_hooded_merganser_Florida_W22-154_2022_H5N1
OP221306_A_bald_eagle_Georgia_W22-194B_2022_H5N1
0.02

d-NP

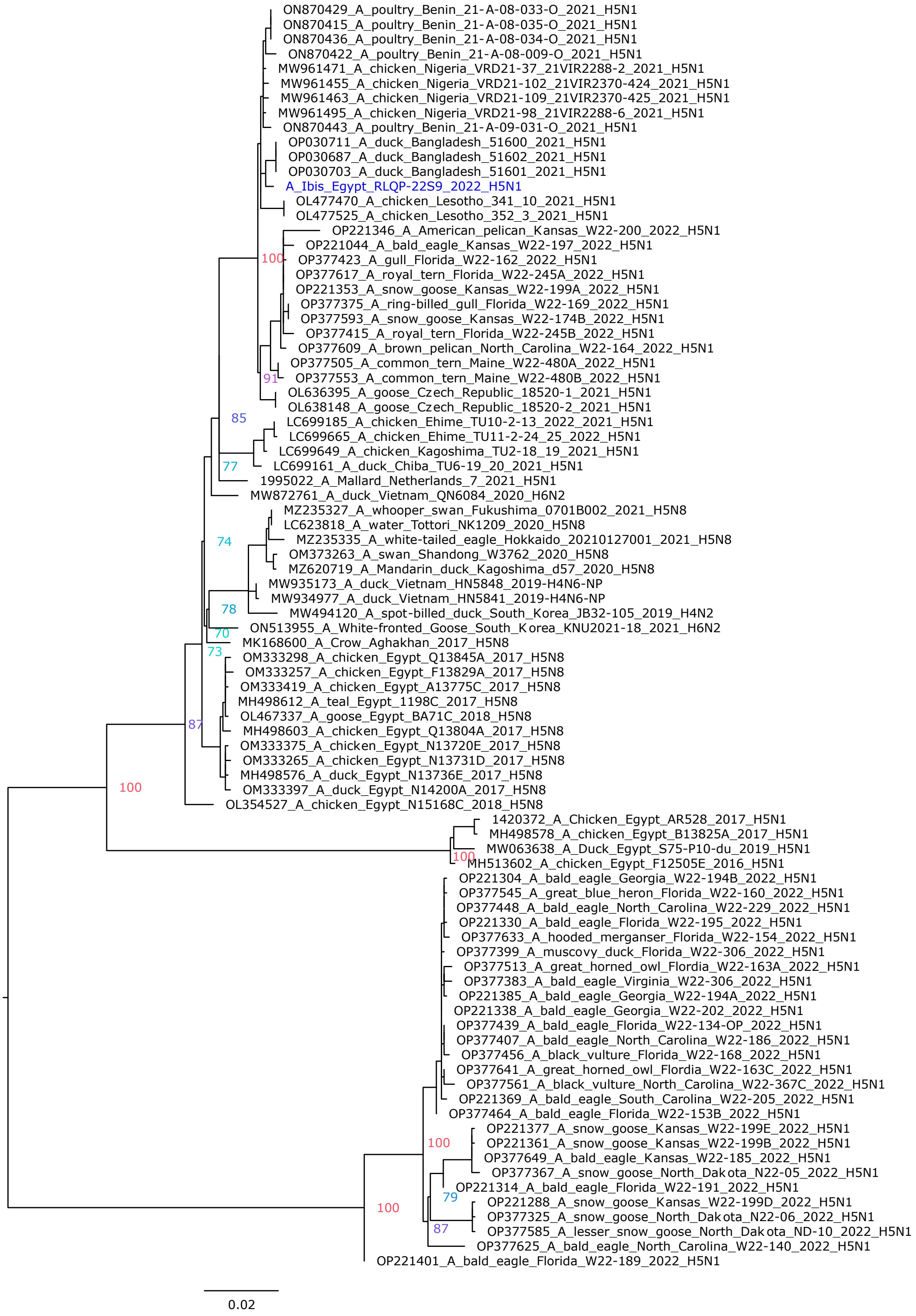
ON870429_A_poultry_Benin_21-A-08-033-O_2021_H5N1
ON870415_A_poultry_Benin_21-A-08-035-O_2021_H5N1
ON870436_A_poultry_Benin_21-A-08-034-O_2021_H5N1
ON870422_A_poultry_Benin_21-A-08-009-O_2021_H5N1
MW961471_A_chicken_Nigeria_VRD21-37_21VIR2288-2_2021_H5N1
MW961455_A_chicken_Nigeria_VRD21-102_21VIR2370-424_2021_H5N1
MW961463_A_chicken_Nigeria_VRD21-109_21VIR2370-425_2021_H5N1
MW961495_A_chicken_Nigeria_VRD21-98_21VIR2288-6_2021_H5N1
ON870443_A_poultry_Benin_21-A-09-031-O_2021_H5N1
OP030711_A_duck_Bangladesh_51600_2021_H5N1
OP030687_A_duck_Bangladesh_51602_2021_H5N1
OP030703_A_duck_Bangladesh_51601_2021_H5N1
A_Ibis_Egypt_RLQP-22S9_2022_H5N1
OL477470_A_chicken_Lesotho_341_10_2021_H5N1
OL477525_A_chicken_Lesotho_352_3_2021_H5N1
OP221346_A_American_pelican_Kansas_W22-200_2022_H5N1
OP221044_A_bald_eagle_Kansas_W22-197_2022_H5N1
100
OP377423_A_gull_Florida_W22-162_2022_H5N1
OP377617_A_royal_tern_Florida_W22-245A_2022_H5N1
OP221353_A_snow_goose_Kansas_W22-199A_2022_H5N1
OP377375_A_ring-billed_gull_Florida_W22-169_2022_H5N1
OP377593_A_snow_goose_Kansas_W22-174B_2022_H5N1
OP377415_A_royal_tern_Florida_W22-245B_2022_H5N1
OP377609_A_brown_pelican_North_Carolina_W22-164_2022_H5N1
OP377505_A_common_tern_Maine_W22-480A_2022_H5N1
91
OP377553_A_common_tern_Maine_W22-480B_2022_H5N1
OL636395_A_goose_Czech_Republic_18520-1_2021_H5N1
OL638148_A_goose_Czech_Republic_18520-2_2021_H5N1
85
LC699185_A_chicken_Ehime_TU10-2-13_2022_2021_H5N1
LC699665_A_chicken_Ehime_TU11-2-24_25_2022_H5N1
LC699649_A_chicken_Kagoshima_TU2-18_19_2021_H5N1
77
LC699161_A_duck_Chiba_TU6-19_20_2021_H5N1
1995022_A_Mallard_Netherlands_7_2021_H5N1
MW872761_A_duck_Vietnam_QN6084_2020_H6N2
MZ235327_A_whooper_swan_Fukushima_0701B002_2021_H5N8
LC623818_A_water_Tottori_NK1209_2020_H5N8
74
MZ235335_A_white-tailed_eagle_Hokkaido_20210127001_2021_H5N8
OM373263_A_swan_Shandong_W3762_2020_H5N8
MZ620719_A_Mandarin_duck_Kagoshima_d57_2020_H5N8
MW935173_A_duck_Vietnam_HN5848_2019-H4N6-NP
MW934977_A_duck_Vietnam_HN5841_2019-H4N6-NP
78
MW494120_A_spot-billed_duck_South_Korea_JB32-105_2019_H4N2
70
ON513955_A_White-fronted_Goose_South_Korea_KNU2021-18_2021_H6N2
MK168600_A_Crow_Aghakhan_2017_H5N8
73
OM333298_A_chicken_Egypt_Q13845A_2017_H5N8
OM333257_A_chicken_Egypt_F13829A_2017_H5N8
OM333419_A_chicken_Egypt_A13775C_2017_H5N8
MH498612_A_teal_Egypt_1198C_2017_H5N8
87
OL467337_A_goose_Egypt_BA71C_2018_H5N8
MH498603_A_chicken_Egypt_Q13804A_2017_H5N8
OM333375_A_chicken_Egypt_N13720E_2017_H5N8
OM333265_A_chicken_Egypt_N13731D_2017_H5N8
MH498576_A_duck_Egypt_N13736E_2017_H5N8
100
OM333397_A_duck_Egypt_N14200A_2017_H5N8
OL354527_A_chicken_Egypt_N15168C_2018_H5N8
1420372_A_Chicken_Egypt_AR528_2017_H5N1
MH498578_A_chicken_Egypt_B13825A_2017_H5N1
MW063638_A_Duck_Egypt_S75-P10-du_2019_H5N1
100
MH513602_A_chicken_Egypt_F12505E_2016_H5N1
OP221304_A_bald_eagle_Georgia_W22-194B_2022_H5N1
OP377545_A_great_blue_heron_Florida_W22-160_2022_H5N1
OP377448_A_bald_eagle_North_Carolina_W22-229_2022_H5N1
OP221330_A_bald_eagle_Florida_W22-195_2022_H5N1
OP377633_A_hooded_merganser_Florida_W22-154_2022_H5N1
OP377399_A_muscovy_duck_Florida_W22-306_2022_H5N1
OP377513_A_great_horned_owl_Flordia_W22-163A_2022_H5N1
OP377383_A_bald_eagle_Virginia_W22-306_2022_H5N1
OP221385_A_bald_eagle_Georgia_W22-194A_2022_H5N1
OP221338_A_bald_eagle_Georgia_W22-202_2022_H5N1
OP377439_A_bald_eagle_Florida_W22-134-OP_2022_H5N1
OP377407_A_bald_eagle_North_Carolina_W22-186_2022_H5N1
OP377456_A_black_vulture_Florida_W22-168_2022_H5N1
OP377641_A_great_horned_owl_Flordia_W22-163C_2022_H5N1
OP377561_A_black_vulture_North_Carolina_W22-367C_2022_H5N1
OP221369_A_bald_eagle_South_Carolina_W22-205_2022_H5N1
OP377464_A_bald_eagle_Florida_W22-153B_2022_H5N1
OP221377_A_snow_goose_Kansas_W22-199E_2022_H5N1
100
OP221361_A_snow_goose_Kansas_W22-199B_2022_H5N1
OP377649_A_bald_eagle_Kansas_W22-185_2022_H5N1
OP377367_A_snow_goose_North_Dakota_N22-05_2022_H5N1
79
OP221314_A_bald_eagle_Florida_W22-191_2022_H5N1
100
OP221288_A_snow_goose_Kansas_W22-199D_2022_H5N1
OP377325_A_snow_goose_North_Dakota_N22-06_2022_H5N1
87
OP377585_A_lesser_snow_goose_North_Dakota_ND-10_2022_H5N1
OP377625_A_bald_eagle_North_Carolina_W22-140_2022_H5N1
OP221401_A_bald_eagle_Florida_W22-189_2022_H5N1
0.02

e-M

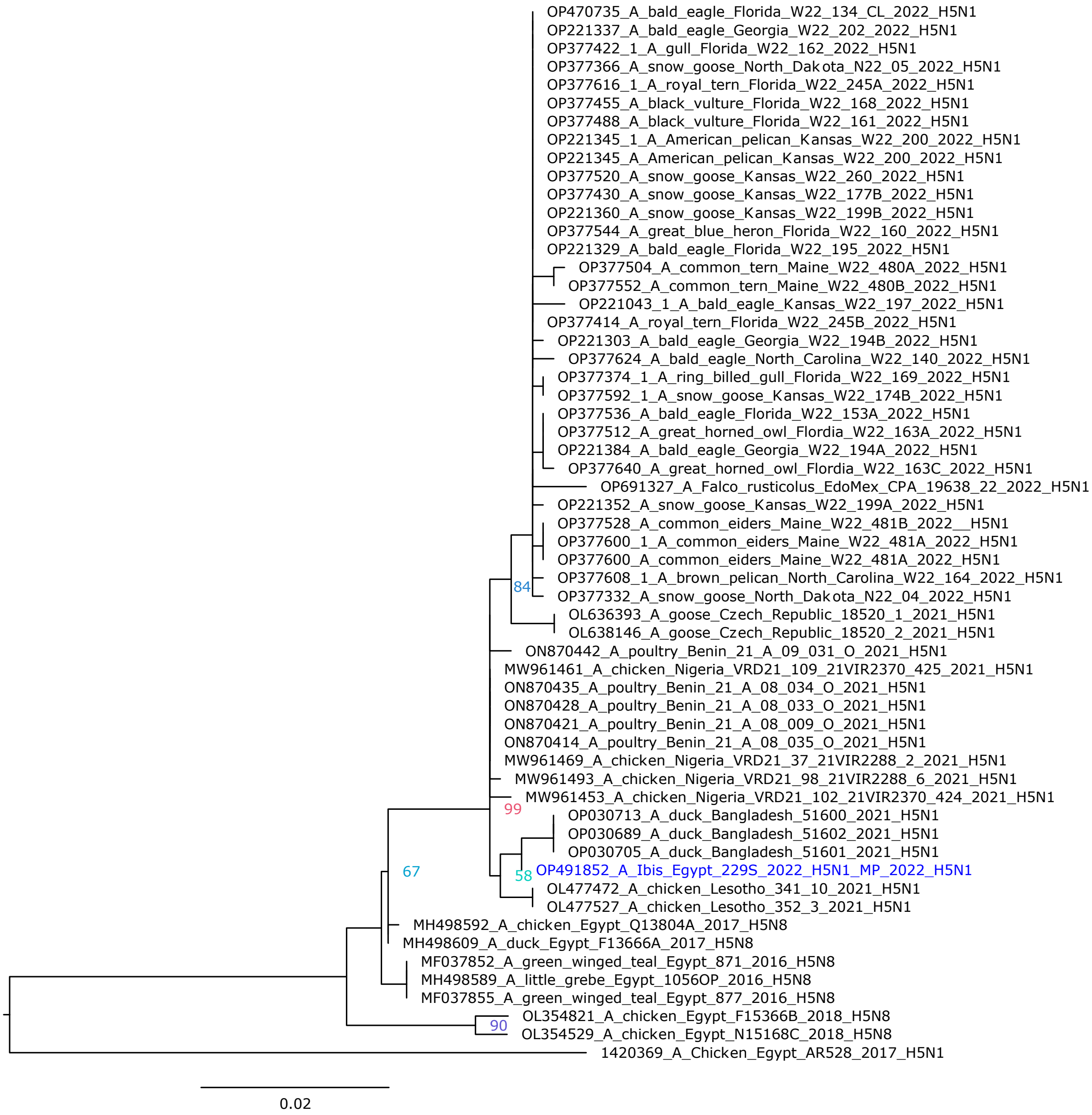

OP470735_A_bald_eagle_Florida_W22_134_CL_2022_H5N1
OP221337_A_bald_eagle_Georgia_W22_202_2022_H5N1
OP377422_1_A_gull_Florida_W22_162_2022_H5N1
OP377366_A_snow_goose_North_Dakota_N22_05_2022_H5N1
OP377616_1_A_royal_tern_Florida_W22_245A_2022_H5N1
OP377455_A_black_vulture_Florida_W22_168_2022_H5N1
OP377488_A_black_vulture_Florida_W22_161_2022_H5N1
OP221345_1_A_American_pelican_Kansas_W22_200_2022_H5N1
OP221345_A_American_pelican_Kansas_W22_200_2022_H5N1
OP377520_A_snow_goose_Kansas_W22_260_2022_H5N1
OP377430_A_snow_goose_Kansas_W22_177B_2022_H5N1
OP221360_A_snow_goose_Kansas_W22_199B_2022_H5N1
OP377544_A_great_blue_heron_Florida_W22_160_2022_H5N1
OP221329_A_bald_eagle_Florida_W22_195_2022_H5N1
OP377504_A_common_tern_Maine_W22_480A_2022_H5N1
OP377552_A_common_tern_Maine_W22_480B_2022_H5N1
OP221043_1_A_bald_eagle_Kansas_W22_197_2022_H5N1
OP377414_A_royal_tern_Florida_W22_245B_2022_H5N1
OP221303_A_bald_eagle_Georgia_W22_194B_2022_H5N1
OP377624_A_bald_eagle_North_Carolina_W22_140_2022_H5N1
OP377374_1_A_ring_billed_gull_Florida_W22_169_2022_H5N1
OP377592_1_A_snow_goose_Kansas_W22_174B_2022_H5N1
OP377536_A_bald_eagle_Florida_W22_153A_2022_H5N1
OP377512_A_great_horned_owl_Flordia_W22_163A_2022_H5N1
OP221384_A_bald_eagle_Georgia_W22_194A_2022_H5N1
OP377640_A_great_horned_owl_Flordia_W22_163C_2022_H5N1
OP691327_A_Falco_rusticolus_EdoMex_CPA_19638_22_2022_H5N1
OP221352_A_snow_goose_Kansas_W22_199A_2022_H5N1
OP377528_A_common_eiders_Maine_W22_481B_2022__H5N1
OP377600_1_A_common_eiders_Maine_W22_481A_2022_H5N1
OP377600_A_common_eiders_Maine_W22_481A_2022_H5N1
84
OP377608_1_A_brown_pelican_North_Carolina_W22_164_2022_H5N1
OP377332_A_snow_goose_North_Dakota_N22_04_2022_H5N1
OL636393_A_goose_Czech_Republic_18520_1_2021_H5N1
OL638146_A_goose_Czech_Republic_18520_2_2021_H5N1
ON870442_A_poultry_Benin_21_A_09_031_O_2021_H5N1
MW961461_A_chicken_Nigeria_VRD21_109_21VIR2370_425_2021_H5N1
ON870435_A_poultry_Benin_21_A_08_034_O_2021_H5N1
ON870428_A_poultry_Benin_21_A_08_033_O_2021_H5N1
ON870421_A_poultry_Benin_21_A_08_009_O_2021_H5N1
ON870414_A_poultry_Benin_21_A_08_035_O_2021_H5N1
MW961469_A_chicken_Nigeria_VRD21_37_21VIR2288_2_2021_H5N1
MW961493_A_chicken_Nigeria_VRD21_98_21VIR2288_6_2021_H5N1
MW961453_A_chicken_Nigeria_VRD21_102_21VIR2370_424_2021_H5N1
99
OP030713_A_duck_Bangladesh_51600_2021_H5N1
OP030689_A_duck_Bangladesh_51602_2021_H5N1
OP030705_A_duck_Bangladesh_51601_2021_H5N1
67
58
OP491852_A_Ibis_Egypt_229S_2022_H5N1_MP_2022_H5N1
OL477472_A_chicken_Lesotho_341_10_2021_H5N1
OL477527_A_chicken_Lesotho_352_3_2021_H5N1
MH498592_A_chicken_Egypt_Q13804A_2017_H5N8
MH498609_A_duck_Egypt_F13666A_2017_H5N8
MF037852_A_green_winged_teal_Egypt_871_2016_H5N8
MH498589_A_little_grebe_Egypt_1056OP_2016_H5N8
MF037855_A_green_winged_teal_Egypt_877_2016_H5N8
90
OL354821_A_chicken_Egypt_F15366B_2018_H5N8
OL354529_A_chicken_Egypt_N15168C_2018_H5N8
1420369_A_Chicken_Egypt_AR528_2017_H5N1
0.02

f-NS

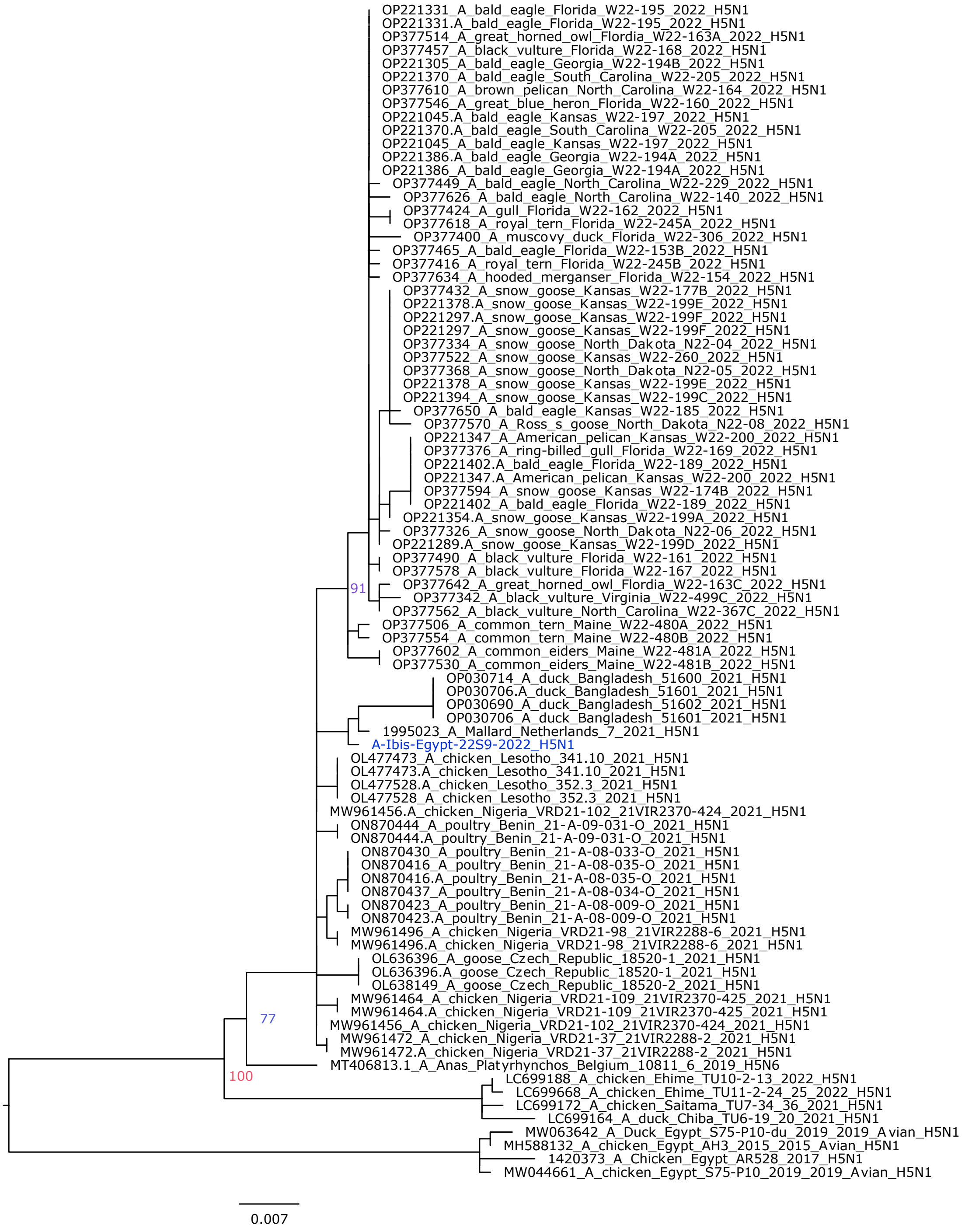

OP221331_A_bald_eagle_Florida_W22-195_2022_H5N1
OP221331.A_bald_eagle_Florida_W22-195_2022_H5N1
OP377514_A_great_horned_owl_Flordia_W22-163A_2022_H5N1
OP377457_A_black_vulture_Florida_W22-168_2022_H5N1
OP221305_A_bald_eagle_Georgia_W22-194B_2022_H5N1
OP221370_A_bald_eagle_South_Carolina_W22-205_2022_H5N1
OP377610_A_brown_pelican_North_Carolina_W22-164_2022_H5N1
OP377546_A_great_blue_heron_Florida_W22-160_2022_H5N1
OP221045.A_bald_eagle_Kansas_W22-197_2022_H5N1
OP221370.A_bald_eagle_South_Carolina_W22-205_2022_H5N1
OP221045_A_bald_eagle_Kansas_W22-197_2022_H5N1
OP221386.A_bald_eagle_Georgia_W22-194A_2022_H5N1
OP221386_A_bald_eagle_Georgia_W22-194A_2022_H5N1
OP377449_A_bald_eagle_North_Carolina_W22-229_2022_H5N1
OP377626_A_bald_eagle_North_Carolina_W22-140_2022_H5N1
OP377424_A_gull_Florida_W22-162_2022_H5N1
OP377618_A_royal_tern_Florida_W22-245A_2022_H5N1
OP377400_A_muscovy_duck_Florida_W22-306_2022_H5N1
OP377465_A_bald_eagle_Florida_W22-153B_2022_H5N1
OP377416_A_royal_tern_Florida_W22-245B_2022_H5N1
OP377634_A_hooded_merganser_Florida_W22-154_2022_H5N1
OP377432_A_snow_goose_Kansas_W22-177B_2022_H5N1
OP221378.A_snow_goose_Kansas_W22-199E_2022_H5N1
OP221297.A_snow_goose_Kansas_W22-199F_2022_H5N1
OP221297_A_snow_goose_Kansas_W22-199F_2022_H5N1
OP377334_A_snow_goose_North_Dakota_N22-04_2022_H5N1
OP377522_A_snow_goose_Kansas_W22-260_2022_H5N1
OP377368_A_snow_goose_North_Dakota_N22-05_2022_H5N1
OP221378_A_snow_goose_Kansas_W22-199E_2022_H5N1
OP221394_A_snow_goose_Kansas_W22-199C_2022_H5N1
OP377650_A_bald_eagle_Kansas_W22-185_2022_H5N1
OP377570_A_Ross_s_goose_North_Dakota_N22-08_2022_H5N1
OP221347_A_American_pelican_Kansas_W22-200_2022_H5N1
OP377376_A_ring-billed_gull_Florida_W22-169_2022_H5N1
OP221402.A_bald_eagle_Florida_W22-189_2022_H5N1
OP221347.A_American_pelican_Kansas_W22-200_2022_H5N1
OP377594_A_snow_goose_Kansas_W22-174B_2022_H5N1
OP221402_A_bald_eagle_Florida_W22-189_2022_H5N1
OP221354.A_snow_goose_Kansas_W22-199A_2022_H5N1
OP377326_A_snow_goose_North_Dakota_N22-06_2022_H5N1
OP221289.A_snow_goose_Kansas_W22-199D_2022_H5N1
OP377490_A_black_vulture_Florida_W22-161_2022_H5N1
OP377578_A_black_vulture_Florida_W22-167_2022_H5N1
91
OP377642_A_great_horned_owl_Flordia_W22-163C_2022_H5N1
OP377342_A_black_vulture_Virginia_W22-499C_2022_H5N1
OP377562_A_black_vulture_North_Carolina_W22-367C_2022_H5N1
OP377506_A_common_tern_Maine_W22-480A_2022_H5N1
OP377554_A_common_tern_Maine_W22-480B_2022_H5N1
OP377602_A_common_eiders_Maine_W22-481A_2022_H5N1
OP377530_A_common_eiders_Maine_W22-481B_2022_H5N1
OP030714_A_duck_Bangladesh_51600_2021_H5N1
OP030706.A_duck_Bangladesh_51601_2021_H5N1
OP030690_A_duck_Bangladesh_51602_2021_H5N1
OP030706_A_duck_Bangladesh_51601_2021_H5N1
1995023_A_Mallard_Netherlands_7_2021_H5N1
A-Ibis-Egypt-22S9-2022_H5N1
OL477473_A_chicken_Lesotho_341.10_2021_H5N1
OL477473.A_chicken_Lesotho_341.10_2021_H5N1
OL477528.A_chicken_Lesotho_352.3_2021_H5N1
OL477528_A_chicken_Lesotho_352.3_2021_H5N1
MW961456.A_chicken_Nigeria_VRD21-102_21VIR2370-424_2021_H5N1
ON870444_A_poultry_Benin_21-A-09-031-O_2021_H5N1
ON870444.A_poultry_Benin_21-A-09-031-O_2021_H5N1
ON870430_A_poultry_Benin_21-A-08-033-O_2021_H5N1
ON870416_A_poultry_Benin_21-A-08-035-O_2021_H5N1
ON870416.A_poultry_Benin_21-A-08-035-O_2021_H5N1
ON870437_A_poultry_Benin_21-A-08-034-O_2021_H5N1
ON870423_A_poultry_Benin_21-A-08-009-O_2021_H5N1
ON870423.A_poultry_Benin_21-A-08-009-O_2021_H5N1
MW961496_A_chicken_Nigeria_VRD21-98_21VIR2288-6_2021_H5N1
MW961496.A_chicken_Nigeria_VRD21-98_21VIR2288-6_2021_H5N1
OL636396_A_goose_Czech_Republic_18520-1_2021_H5N1
OL636396.A_goose_Czech_Republic_18520-1_2021_H5N1
OL638149_A_goose_Czech_Republic_18520-2_2021_H5N1
MW961464_A_chicken_Nigeria_VRD21-109_21VIR2370-425_2021_H5N1
MW961464.A_chicken_Nigeria_VRD21-109_21VIR2370-425_2021_H5N1
77
MW961456_A_chicken_Nigeria_VRD21-102_21VIR2370-424_2021_H5N1
MW961472_A_chicken_Nigeria_VRD21-37_21VIR2288-2_2021_H5N1
MW961472.A_chicken_Nigeria_VRD21-37_21VIR2288-2_2021_H5N1
MT406813.1_A_Anas_Platyrhynchos_Belgium_10811_6_2019_H5N6
100
LC699188_A_chicken_Ehime_TU10-2-13_2022_H5N1
LC699668_A_chicken_Ehime_TU11-2-24_25_2022_H5N1
LC699172_A_chicken_Saitama_TU7-34_36_2021_H5N1
LC699164_A_duck_Chiba_TU6-19_20_2021_H5N1
MW063642_A_Duck_Egypt_S75-P10-du_2019_2019_Avian_H5N1
MH588132_A_chicken_Egypt_AH3_2015_2015_Avian_H5N1
1420373_A_Chicken_Egypt_AR528_2017_H5N1
MW044661_A_chicken_Egypt_S75-P10_2019_2019_Avian_H5N1
0.007

Reference:

1. Spackman, E.; Senne, D.A.; Myers, T.; Bulaga, L.L.; Garber, L.P.; Perdue, M.L.; Lohman, K.; Daum, L.T.; Suarez, D.L. Development of a real-time reverse transcriptase PCR assay for type A influenza virus and the avian H5 and H7 hemagglutinin subtypes. *Journal of clinical microbiology* **2002**, *40*, 3256-3260.
2. Slomka, M.; Pavlidis, T.; Banks, J.; Shell, W.; McNally, A.; Essen, S.; Brown, I. Validated H5 Eurasian real-time reverse transcriptase–polymerase chain reaction and its application in H5N1 outbreaks in 2005–2006. *Avian diseases* **2007**, *51*, 373-377.
3. Hoffmann, B.; Hoffmann, D.; Henritzi, D.; Beer, M.; Harder, T.C. Riems influenza a typing array (RITA): An RT-qPCR-based low density array for subtyping avian and mammalian influenza a viruses. *Scientific reports* **2016**, *6*, 1-10.
4. Ben Shabat, M.; Meir, R.; Haddas, R.; Lapin, E.; Shkoda, I.; Raibstein, I.; Perk, S.; Davidson, I. Development of a real-time TaqMan RT-PCR assay for the detection of H9N2 avian influenza viruses. *Journal of virological methods* **2010**, *168*, 72-77, doi:10.1016/j.jviromet.2010.04.019.
5. Meir, R.; Maharat, O.; Farnushi, Y.; Simanov, L. Development of a real-time TaqMan® RT-PCR assay for the detection of infectious bronchitis virus in chickens, and comparison of RT-PCR and virus isolation. *Journal of virological methods* **2010**, *163*, 190-194.
6. Wise, M.G.; Suarez, D.L.; Seal, B.S.; Pedersen, J.C.; Senne, D.A.; King, D.J.; Kapczynski, D.R.; Spackman, E. Development of a real-time reverse-transcription PCR for detection of Newcastle disease virus RNA in clinical samples. *Journal of Clinical Microbiology* **2004**, *42*, 329-338.
7. Mo, J.; Angelichio, M.; Gow, L.; Leathers, V.; Jackwood, M.W. Quantitative real-time PCR assays for the concurrent diagnosis of infectious laryngotracheitis virus, Newcastle disease virus and avian metapneumovirus in poultry. *Journal of Veterinary Science* **2022**, *23*.
8. Herman, N. Real-Time Rt-Pcr Assays To Detect West Nile Virus In Mosquito Pools. Yale University, 2015.
9. Metwally, A.; Yousif, A.; Shaheed, I.; Mohammed, W.; Samy, A.; Reda, I. Re-emergence of very virulent IBDV in Egypt. *International journal of virology* **2009**, *5*, 1-17.
10. Hoffmann, E.; Stech, J.; Guan, Y.; Webster, R.; Perez, D. Universal primer set for the full-length amplification of all influenza A viruses. *Archives of virology* **2001**, *146*, 2275-2289.
11. Mady, W.; Yehia, N.; Elhussieny, M.H.; Shalaby, A.G.; El Sayed, M.M.; Bakry, N.R.; Shaaban, A.; Arafa, A.S.; Mahmoud, A.A.B.; Hassan, W.M. Genetic characterization of highly pathogenic avian influenza virus (H5N8) in backyard poultry production sector during Mid-2017 to autumn 2018. *Hosts and Viruses* **2019**, *6*, 75.
